# Rapid production of antigen-specific monoclonal antibodies from a variety of animals

**DOI:** 10.1186/1741-7007-10-80

**Published:** 2012-09-28

**Authors:** Nobuyuki Kurosawa, Megumi Yoshioka, Rika Fujimoto, Fuminori Yamagishi, Masaharu Isobe

**Affiliations:** 1 Laboratory of Molecular and Cellular Biology, Faculty of Science and Engineering, Graduate School, University of Toyama, 3190 Gofuku, Toyama-shi, Toyama, 930-8555, Japan; 2 Graduate School of Innovative Life Science, University of Toyama, Toyama-shi, Toyama, 930-8555, Japan; 3 Department of Immunology, Kochi Medical School, Nangoku-shi, Kochi, 783-8505, Japan; 4 Department of Surgery, Itoigawa General Hospital, Itoigawa-shi, Niigata, 941-0006, Japan

**Keywords:** antigen-specific monoclonal antibody, ER-tracker, ERIAA, FACS, guinea pig, human, MAGrahd, rabbit, rat, single cell, TS-jPCR

## Abstract

**Background:**

Although a variety of animals have been used to produce polyclonal antibodies against antigens, the production of antigen-specific monoclonal antibodies from animals remains challenging.

**Results:**

We propose a simple and rapid strategy to produce monoclonal antibodies from a variety of animals. By staining lymph node cells with an antibody against immunoglobulin and a fluorescent dye specific for the endoplasmic reticulum, plasma/plasmablast cells were identified without using a series of antibodies against lineage markers. By using a fluorescently labeled antigen as a tag for a complementary cell surface immunoglobulin, antigen-specific plasma/plasmablast cells were sorted from the rest of the cell population by fluorescence-activated cell sorting. Amplification of cognate pairs of immunoglobulin heavy and light chain genes followed by DNA transfection into 293FT cells resulted in the highly efficient production of antigen-specific monoclonal antibodies from a variety of immunized animals.

**Conclusions:**

Our technology eliminates the need for both cell propagation and screening processes, offering a significant advantage over hybridoma and display strategies.

## Background

The mouse hybridoma method has been used previously for the production of candidate monoclonal antibodies (mAbs) for therapeutic use [1]. However, immune responses against highly conserved human proteins are often weak in mice, resulting in the production of low affinity and/or non-specific mAbs. To avoid the problem of human proteins being recognized as self-antigens in mice, the use of an evolutionarily distant animal from humans is essential to obtain better immunization against therapeutic target molecules. While a variety of animals have been used to produce polyclonal antibodies against human proteins, mAbs from animals other than rodents have not been routinely produced due to the difficulties in establishing immortalized antibody-producing cell lines by hybridoma, viral transformation or reprogramming [1-3]. Recently, the direct molecular cloning of cognate pairs of immunoglobulin gamma heavy chain (IgH) variable (V_H_), light chain kappa variable (V_Lκ_) and light chain lambda variable (V_Lλ_) genes from single antigen-specific plasma/plasmablast cells (ASPCs) using the polymerase chain reaction (PCR) has attracted attention as an alternative method for generating mAbs from immunized animals [4-7]. Although the use of ASPCs is best suited to the isolation of high affinity mAbs, since they go through the processes of somatic hypermutation and affinity maturation, the application of this method for species other than humans and mice is limited because the current plasma/plasmablast cell (PC) isolation protocols rely on a small number of identified PC-specific markers combined with the absence of one or more B cell differentiation antigens [8]. Furthermore, expensive equipment and acquired technical skills are required to identify and isolate ASPCs from the bulk of the PC population [5]. The manual V_H _and V_L _gene amplification from single cells followed by the construction of IgH and immunoglobulin light chain (IgL) gene expression plasmids are also limiting steps of this method [4,9-12]. To achieve rapid and scalable automation for the generation of mAbs from a large numbers of single cells, we previously proposed a high-throughput single-cell-based immunoglobulin gene cloning method by developing a non-contact magnetic power transmission system (MAGrahd) for single-cell-based cDNA synthesis and a target-selective joint PCR (TS-jPCR) for IgH and IgL gene expression [13,14].

**Figure 1:**
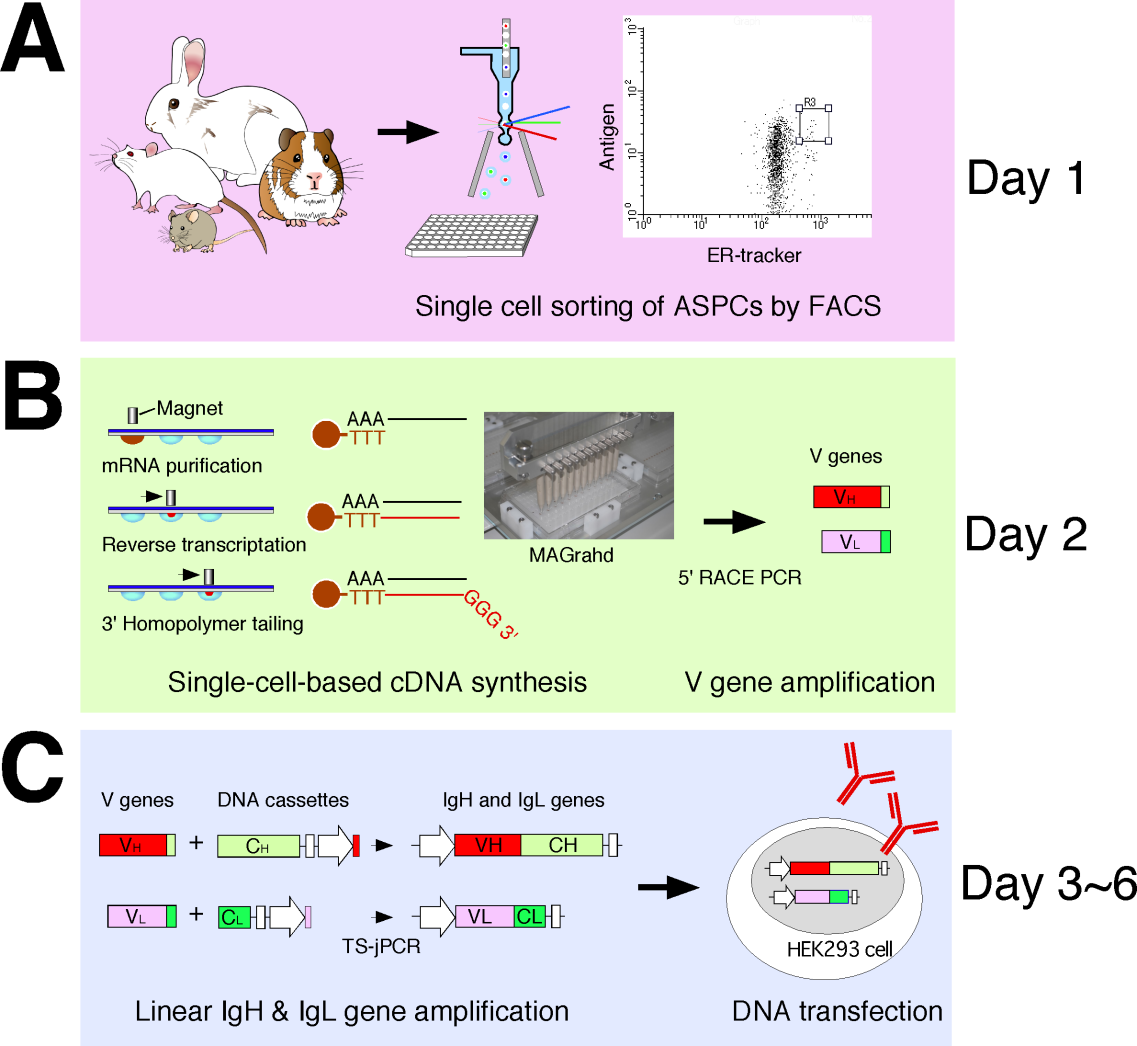
**Flowchart summarizing the generation of antigen-specific monoclonal antibodies (mAbs) from a variety of animals**. **(A) **Fluorescence-activated cell sorting (FACS)-based antigen-specific plasma/plasmablast cell (ASPC) isolation from a variety of animals. Lymphocytes were stained with fluorescently labeled antibodies against IgG, ER-tracker and fluorescently labeled antigen. ASPCs defined as IgG^low ^endoplasmic reticulum (ER)^high ^antigen^+ ^were single-cell sorted into individual wells of 96-well plates. **(B) **Amplification of cognate pairs of V_H _and V_L _genes from single-cell-sorted ASPCs. Preparation of 3'-end homopolymer-tailed cDNA from single-cell-sorted ASPCs was performed automatically by MAGrahd. Cognate pairs of V_H _and V_L _genes were amplified by 5' rapid amplification of cDNA ends (RACE) polymerase chain reaction (PCR). **(C) **Expression of recombinant mAbs. The amplified V_H _and V_L _genes were selectively assembled with IgH and IgL DNA cassettes by target-selective joint PCR (TS-jPCR) to generate linear IgH and IgL genes. The pairs of IgH and IgL genes were cotransfected into 293FT cells grown in 96-well culture dishes.

Although fluorescence-activated cell sorting (FACS) has been used to enrich for particular cell types, there is no single method to achieve a high yield of isolated ASPCs with high purity [15-18]. The main limitation with FACS has been the use of multiple antibodies against lineage-specific markers to enrich for PCs, causing cell membrane damage that produces background noise when identifying ASPCs with fluorescently labeled antigens. In this paper, we develop a new antigen-specific PC screening method, termed endoplasmic reticulum (ER)-based identification of antigen-specific antibody-producing cells (ERIAA), using FACS and a fluorescent dye specific for the endoplasmic reticulum. By combining ERIAA and our previously proposed high-throughput single-cell-based Ig gene cloning method, we have created a 1-week protocol for the production of recombinant mAbs from a variety of immunized animals (Figure 1).

## Results

### PC identification without using antibodies against lineage markers

**Figure 2:**
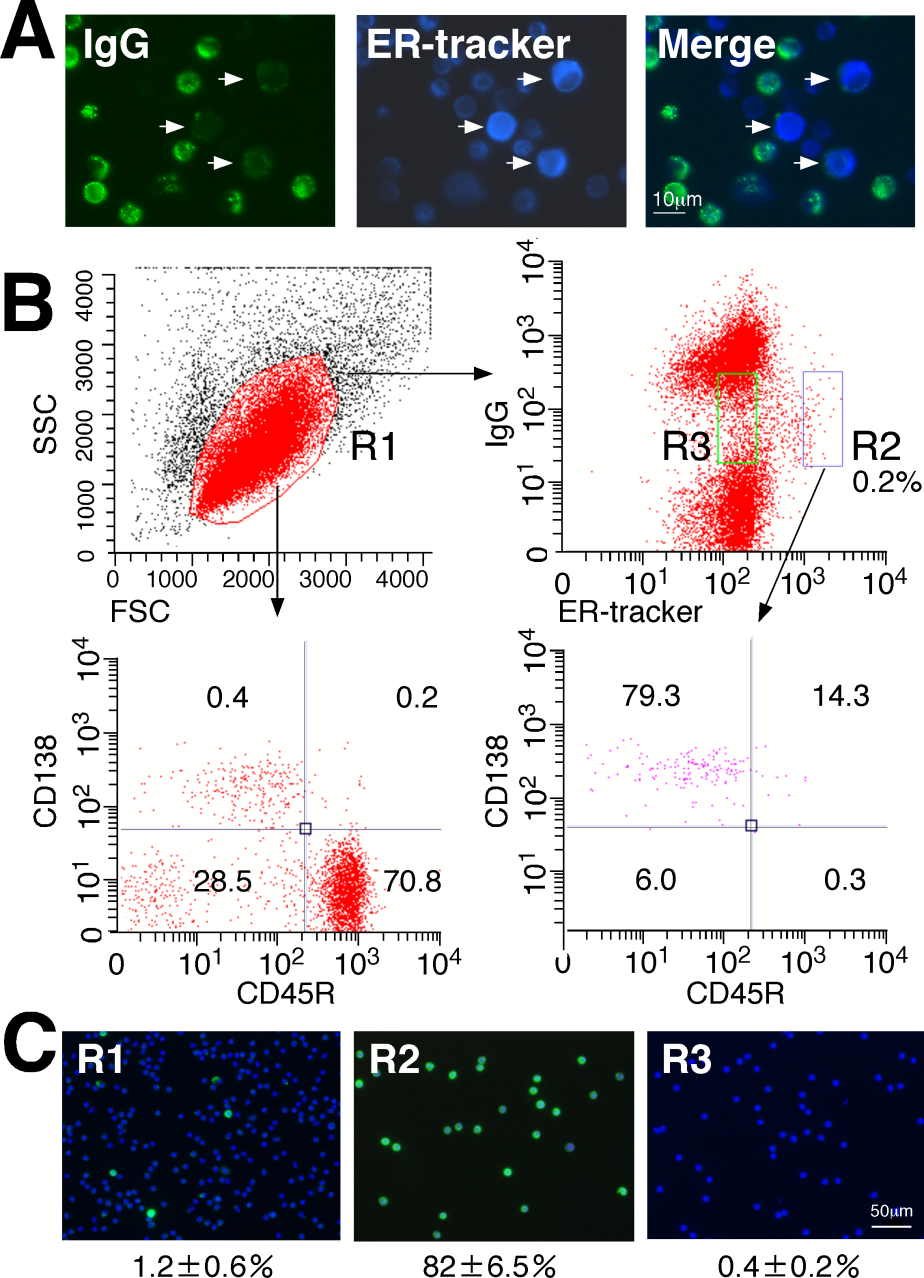
**Identification of mouse plasma/plasmablast cells (PCs) with a fluorescent dye specific for the endoplasmic reticulum (ER)**. **(A) **Cytology images of mouse lymph node cells stained with anti-mouse IgG (green) and ER-tracker (blue). The arrow indicates the IgG^low ^ER^high ^cells. **(B) **A representative fluorescence-activated cell sorting (FACS) graph of cells prepared from the lymph nodes of egg albumin-immunized mice. The forward-versus-side-scatter (FSC vs SSC) with gate R1 represents lymphocytes. The R1-gated cells were stained with anti-mouse IgG Dylight 488 and ER-tracker, and the IgG^low ^ER^high ^cells (R2) were further analyzed for CD38-APC versus CD45R-PE surface expression using FACS. The relative number of cells in each region is given as a mean percentage of three separate experiments. **(C) **FACS-sorted R1-gated, R2-gated and R3-gated cells stained intracellularly with anti-mouse IgG Dylight 488 (green) and 4',6-diamidino-2-phenylindole (DAPI; blue). The numbers indicate the mean percentages of the cells with intense cytoplasmic IgG from three separate experiments.

**Figure 3:**
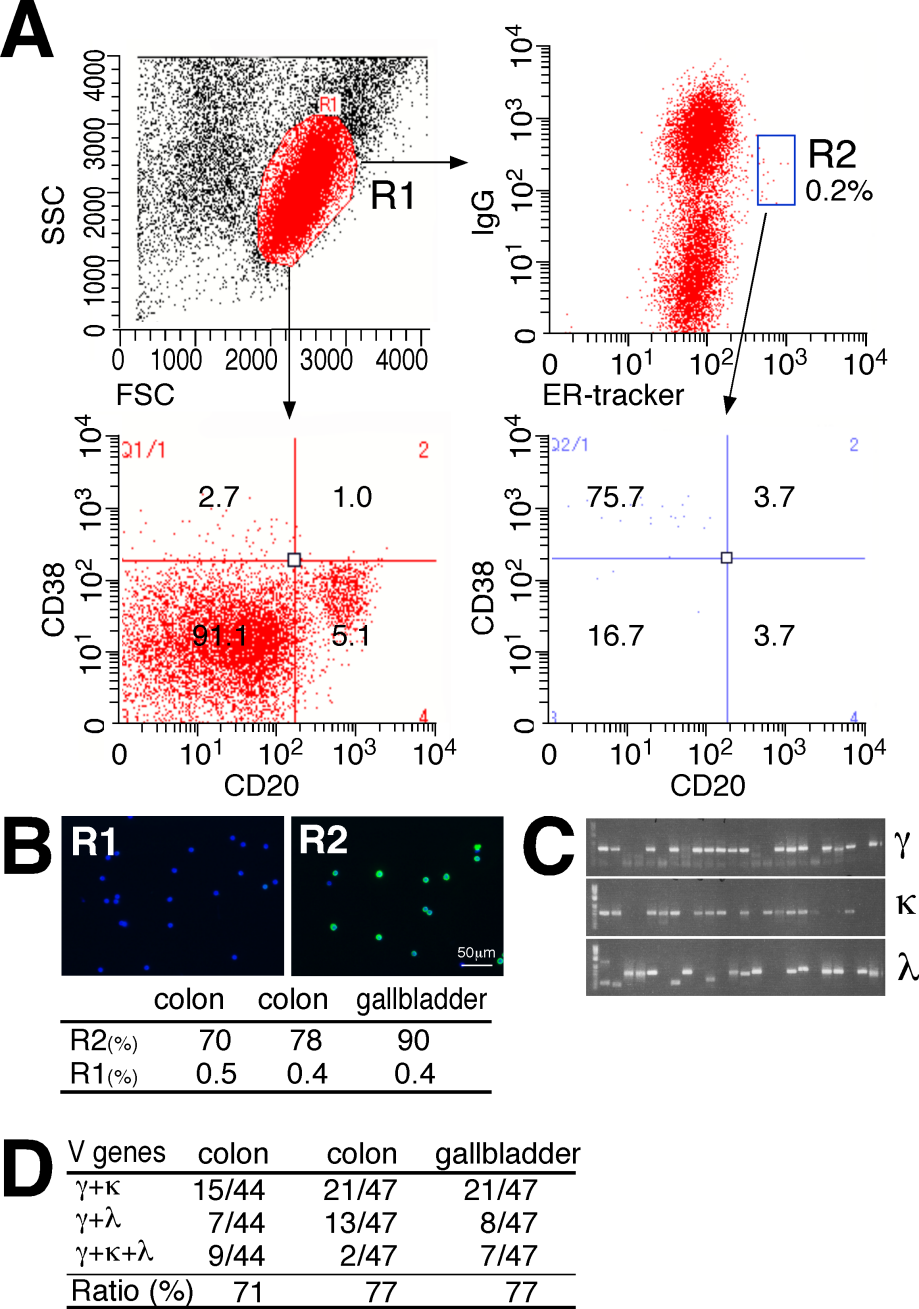
**Human plasma/plasmablast cell (PC) isolation by fluorescence-activated cell sorting (FACS) using ER-tracker and anti-human IgG**. **(A) **A representative FACS graph of cells prepared from the lymph nodes of cancer patients. Cells were stained with anti-human IgG Dylight 488 and ER-tracker, and the IgG^low ^endoplasmic reticulum (ER)^high ^cells were further analyzed for CD38-APC versus CD20-PE surface expression using FACS. The forward-versus-side-scatter (FSC vs SSC) with gate R1 represents lymphocytes. PCs are defined herein as CD38^high ^CD20^low ^cells. A representative FACS graph of three separate experiments is shown. The relative number of cells in each region is given as a mean percentage of three separate experiments. **(B) **R1-gated and R2-gated cells stained intracellularly with anti-human IgG Dylight 488 (green). Nuclei are stained with 4',6-diamidino-2-phenylindole (DAPI; blue). The numbers indicate the percentages of cells with intense cytoplasmic IgG. **(C) **Representative agarose gel electrophoresis of cognate pairs of V genes amplified from single-cell-sorted R3-gated cells. **(D) **Polymerase chain reaction (PCR) success ratio for V genes from single-cell-sorted R3-gated cells from lymph nodes of patients with colon and gallbladder cancers.

PCs contain abundant ER, reflecting the commitment to producing large amounts of secreted antibodies. Thus, we hypothesized that a fluorescent dye specific for the ER could be used to identify PCs. Lymph node cells from egg albumin-immunized mice were stained with ER-Tracker™ Blue-White DPX dye (Life Technologies (http://www.lifetechnologies.com/), hereafter referred to as ER-tracker) and anti-mouse IgG Dylight 488 (Abcam (http://www.abcam.com/)) to identify cells with intense cytoplasmic ER-derived fluorescence and weak cell surface IgG expression (Figure 2A). FACS analysis of the lymphocytes (R1 gate) with anti-mouse IgG Dylight 488 and ER-tracker, combined with anti-CD38-APC and CD45R-PE, revealed that 79.3% of the IgG^low ^ER^high ^cells (R2 gate) were found in the PC fraction, which is characterized as being CD138^high ^CD45R^low ^(Figure 2B). R2-gated cells were analyzed for their expression of cytoplasmic IgG by their morphologic appearance, which was examined by immunofluorescence staining after FACS sorting. An intense cytoplasmic IgG signal was found in 82% of the R2-gated cells, representing a 68-fold enrichment of PCs compared with the original lymphocyte populations (R1 gate); however, the IgG^low ^ER^low ^cells (R3 gate) contained only 0.4% PCs (Figure 2C). Using this procedure, we also tried to isolate human PCs from the lymph nodes of cancer patients (Figure 3). FACS analysis of the lymphocytes (R1 gate) with anti-human IgG Dylight 488 and ER-tracker combined with anti-CD38-APC and CD20-PE revealed that 75.7% of the IgG^low ^ER^high ^cells (R2 gate) were found in the PC fraction. After R2-gated cells were sorted by FACS, their capacity to express abundant cytoplasmic IgG was examined by immunofluorescence staining. An intense cytoplasmic IgG signal was found in 70% to 90% of the R2-gated cells, which represented a 143-fold to 223-fold enrichment of PCs compared with the original lymphocyte population (R1 gate) containing only 0.4% to 0.5% PCs. The percentage of PCs identified by IgG^low ^ER^high ^(0.2%) in R2 gate is not compatible to that identified by lineage markers (2.7%). Given that the percentage of PCs determined by immunofluorescence staining of cytosolic IgG was only 0.4% to 0.5%, PC separation with surface IgG and ER-tracker is more specific than that with two lineage markers. Rapid amplification of 5' cDNA ends PCR (5' RACE PCR) of the single-cell-sorted R2-gated cells resulted in the amplification of cognate pairs of V_H_, V_Lκ _and/or V_Lλ _genes, with a success ratio of 71% to 77%. These results clearly indicate that PCs can be isolated without using antibodies against lineage markers.

### Isolation of ASPCs by FACS

**Figure 4:**
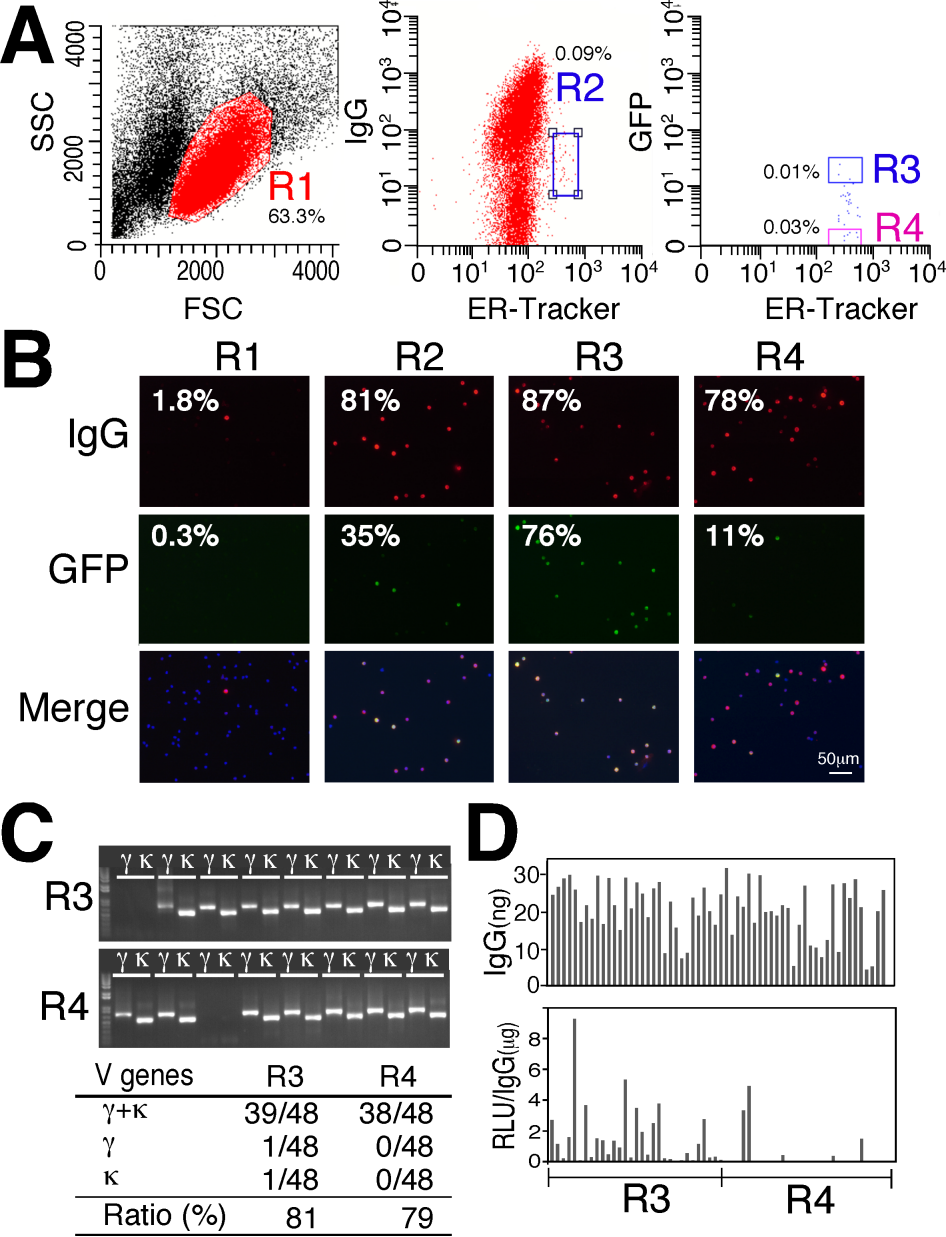
**Isolation of rabbit antigen-specific plasma/plasmablast cells (ASPCs) using fluorescence-activated cell sorting (FACS)**. **(A) **A representative FACS graph of the lymphocytes of green fluorescent protein (GFP)-immunized rabbits stained with anti-rabbit IgG Dylight 650, ER-tracker and GFP Dylight 488. The forward-versus-side-scatter (FSC vs SSC) with gate R1 represents lymphocytes. Plasma/plasmablast cells (PCs) were gated as IgG^low ^endoplasmic reticulum (ER)^high ^(R2). The R2-gated cells were further subdivided into the ASPCs (IgG^low ^ER^high ^GFP^+^, R3 gate) and non-specific PCs (IgG^low ^ER^high ^GFP^-^, R4 gate). The numbers indicate the mean percentages of cells in the gated area from three separate experiments. **(B) **R1-gated, R2-gated, R3-gated and R4-gated cells stained intracellularly with anti-rabbit IgG Dylight 594 (red) and GFP-Dylight 488 (green). The numbers indicate the mean percentages of cells exhibiting IgG and GFP signals from two separate experiments. **(C) **A representative agarose gel electrophoresis of cognate pairs of V genes amplified from single-cell-sorted R3-gated and R4-gated cells (upper). Polymerase chain reaction (PCR) success ratio for V genes from single-cell-sorted R3-gated and R4-gated cells (lower). **(D) **The antigen specificity of rabbit monoclonal antibodies (mAbs) produced from R3-gated and R4-gated cells. Cognate pairs of linear IgH and IgL genes were cotransfected into 293FT cells, and the concentration of antibodies in the cell culture supernatant was determined (upper). The antigen specificity of the mAbs was expressed as relative light units (RLU)/IgG (lower).

**Figure 5:**
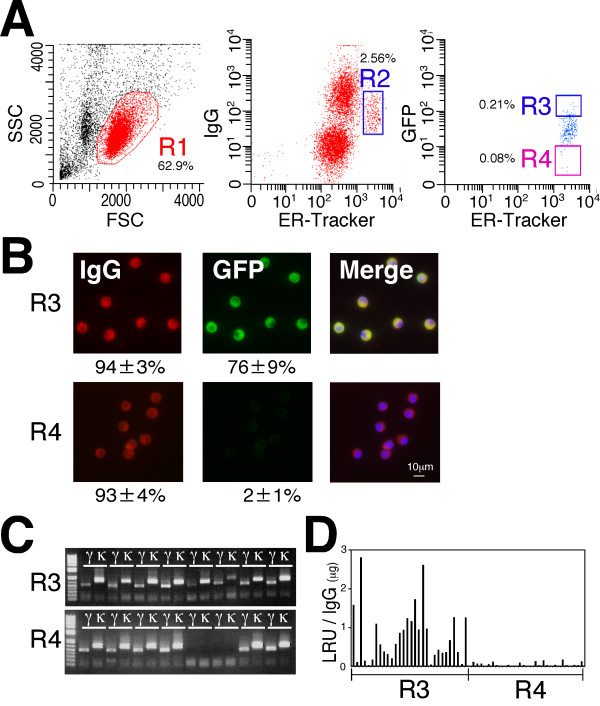
**Isolation of rat antigen-specific plasma/plasmablast cells (ASPCs) using fluorescence-activated cell sorting (FACS)**. **(A) **A representative FACS graph of the lymphocytes from green fluorescent protein (GFP)-immunized rats stained with anti-rat IgG Dylight 650, ER-tracker and GFP-Dylight 488. The forward-versus-side-scatter (FSC vs SSC) with gate R1 represents lymphocytes. Plasma/plasmablast cell (PCs) were gated as IgG^low ^endoplasmic reticulum (ER)^high ^(R2). The R2-gated cells were further subdivided into the ASPCs (IgG^low ^ER^high ^GFP^+^, R3 gate) and non-specific PCs (IgG^low ^ER^high ^GFP^-^, R4 gate). The numbers indicate the mean percentages of cells in the gated area from three separate experiments. **(B) **The R3-gated and R4-gated cells stained intracellularly with anti-rat IgG Dylight 594 (red) and GFP Dylight 488 (green). The numbers indicate the mean percentages of cells with IgG and GFP signals from three separate experiments. **(C) **Representative agarose gel electrophoresis of cognate pairs of V genes amplified from single-cell-sorted R3-gated and R4-gated cells. **(D) **Antigen specificity of the rat monoclonal antibodies (mAbs) produced from R3-gated and R4-gated cells. Cognate pairs of linear IgH and IgL genes were cotransfected into 293FT cells. The antigen specificity of the mAbs was expressed as relative light units (RLU)/IgG.

The advantage of using the ER-tracker is that a series of antibodies against lineage markers is not required for PC identification. Therefore, this procedure enables PC isolation from a variety of animals and can also dramatically reduce cell damage, which in turn will decrease the non-specific binding of labeled antigens to the cell surface. Thus, we next attempted to isolate ASPCs using a fluorescently labeled antigen as a tag for a complementary cell surface IgG and to sort the cells from the rest of the cell population using FACS. Green fluorescent protein (GFP) was selected as a model foreign antigen against immunized rabbits, which were chosen for their robust immune system compared to mice and other rodents. FACS analysis of the lymphocytes of the GFP-immunized rabbits (R1 gate) with anti-rabbit IgG Dylight 650 (Abcam), ER-tracker and GFP Dylight 488 revealed the presence of a PC fraction (defined as IgG^low ^ER^high^) (R2 gate). The cells in the R2 gate were further subdivided into fractions according to their binding levels of GFP to define the ASPCs (IgG^low ^ER^high ^GFP^+^, R3 gate) and non-specific PCs (IgG^low ^ER^high ^GFP^-^, R4 gate) (Figure 4A). We expected the R3-gated cells to contain GFP-specific IgG in the cytoplasm. To test this hypothesis, the cells in the R3 and R4 gates were sorted and examined for expression of an IgG specific for GFP by immunofluorescence staining. In all, 76% of R3-gated cells were found to express an IgG specific for GFP, which was a 252-fold enrichment of ASPCs compared to the original lymphocyte population (R1 gate). Although 78% of the IgG^low ^ER^high ^GFP^- ^cells (R4 gate) expressed an intense cytoplasmic IgG signal, only 11% of the cells reacted with GFP (Figure 4B). A single-cell-based 5' RACE PCR of the R3-gated and R4-gated cells resulted in the amplification of cognate pairs of V_H _and V_Lκ _genes, with an overall success rate of 81% and 79%, respectively (Figure 4C). When pairs of linear IgH and IgL genes were prepared from each pair of V_H _and V_Lκ _genes by TS-jPCR and transfected into 293FT cells, mAbs specific for GFP were produced with a high efficiency from the R3-gated cells (Figure 4D). To determine if this FACS-based screening method, termed ERIAA, can be applied to other animals, we also evaluated ASPC isolation using GFP-immunized rats (Figure 5). FACS analysis of the rat lymphocytes showed the presence of PC cells (R2 gate), and the R2-gated cells were further classified based on their reactivity with GFP as either IgG^low ^ER^high ^GFP^+ ^cells (R3) or IgG^low ^ER^high ^GFP^- ^cells (R4). Our immunofluorescence analysis of the R3-gated cells demonstrated that 76% of the cells reacted with GFP, whereas only 2% of the R4-gated cells reacted with GFP. Although both rabbits and rats received the same GFP as the immunogen, rats had higher PC fraction than that of rabbits (2.56% versus 0.09%). This is probably due to the differences in antigen recognition between the two animals. Amplification of cognate pairs of V_H _and V_Lκ _genes followed by DNA transfection of pairs of linear IgH and IgL genes into 293FT cells resulted in the highly efficient production of GFP-specific antibodies by the R3-gated cells but not by the R4-gated cells. These results clearly demonstrate that ERIAA provides a powerful method to identify and collect ASPCs from a variety of animals in a rapid one-step process.

### Production of guinea pig mAbs against human insulin

**Figure 6:**
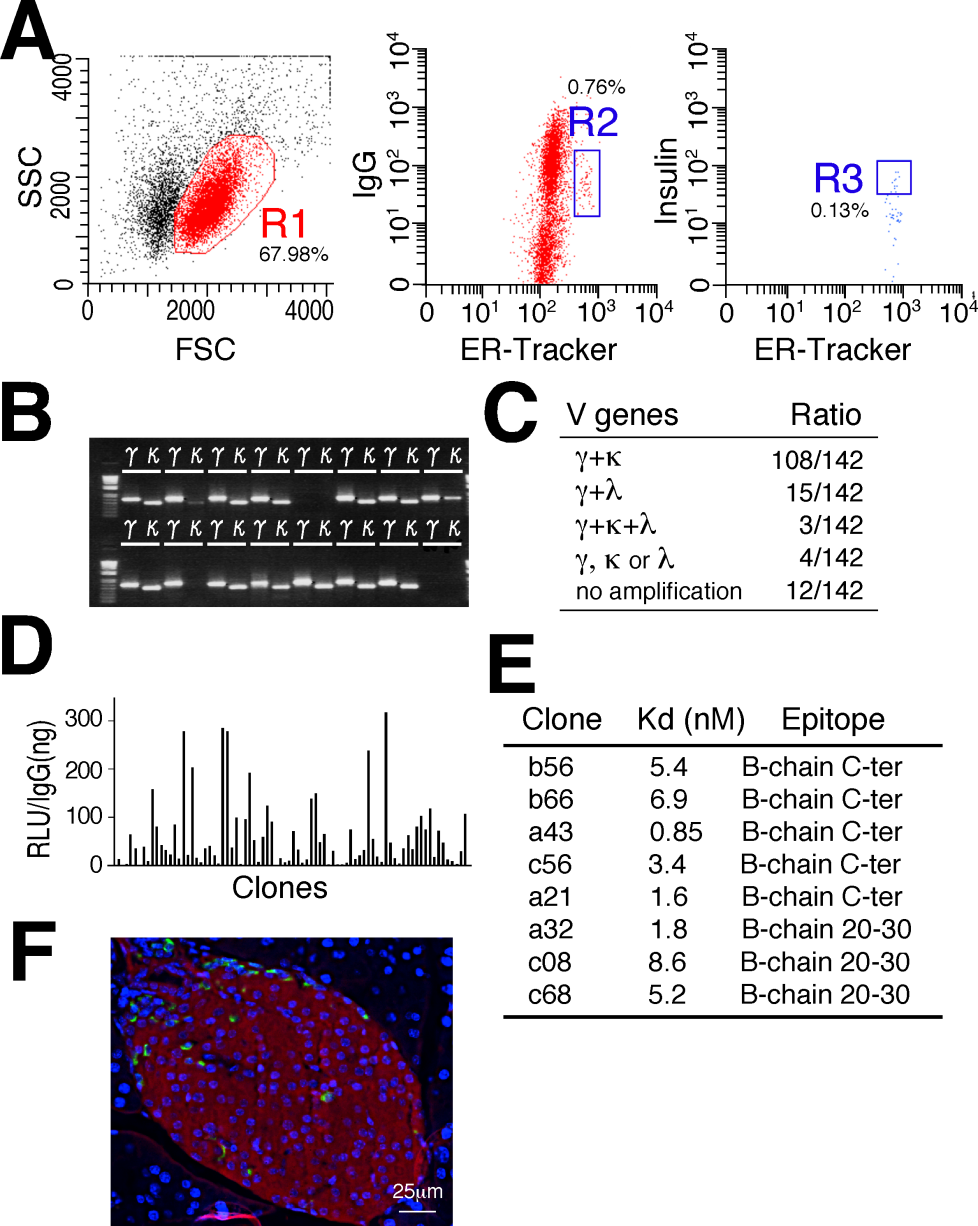
**Production of guinea pig monoclonal antibodies (mAbs) against human insulin**. **(A) **Representative fluorescence-activated cell sorting (FACS) graph of human insulin-immunized guinea pig lymphocytes stained with anti-guinea pig IgG, ER-tracker and insulin-Cy3. Gates for lymphocytes (R1), plasma/plasmablast cells (PCs) (IgG^low ^endoplasmic reticulum (ER)^high^, R2) and antigen-specific plasma/plasmablast cells (ASPCs) (IgG^low ^ER^high ^insulin^+^, R3) were established. **(B) **Representative agarose gel electrophoresis of cognate pairs of V genes amplified from single-cell-sorted R3-gated cells. **(C) **polymerase chain reaction (PCR) success ratio for the V genes. **(D) **Antigen specificity of mAbs produced from the R3-gated cells. **(E) **Binding affinity and binding epitopes of the highly binding clones in (D). **(F) **Immunohistochemical staining of insulin with c08 mAb in (E) (red) and glucagon (green) in a mouse pancreatic section.

Because antibodies in animals are produced by their immune systems, a strong antibody response to a given antigen occurs when there is a wide phylogenetic distance between the immunized animal and the species providing the antigen. Mature human insulin differs from guinea pig insulin at 18 of 51 amino acids but differs from rat and mice insulin at only 4 residues. Therefore, guinea pigs produce high-affinity antibodies against human insulin. Because the introduction of guinea pig mAbs in insulin immunoassays may reduce the amount of undesirable variables caused by the use of polyclonal antibodies, we sought to generate a guinea pig mAb against human insulin. Lymphocytes from human insulin-immunized guinea pigs were stained with anti-guinea pig IgG Dylight 488, ER-tracker and insulin-Cy3, and the insulin-specific PCs were gated as IgG^low ^ER^high ^insulin^+ ^(R3 gate) (Figure 6A). A high amplification success rate for cognate pairs of V_H_, V_Lκ _and/or V_Lλ _genes from the single-cell-sorted R3-gated cells was achieved (89%, 126 of 142) (Figure 6B,C). DNA transfection of pairs of the linear IgH and IgL genes into 293FT cells resulted in the highly efficient production of recombinant guinea pig mAbs against human insulin (Figure 6D). When eight of the highly binding clones were selected for large-scale expression and their dissociation constants were analyzed, they demonstrated high affinities (K_d _= 0.85 to 8.6 nM) (Figure 6E and Additional file 1). DNA sequencing of these clones revealed that the mAbs were divided into three phylogenetic clusters (Additional file 2). Western blot and peptide competition analyses revealed that clones clustered in group 1 (a32, c08 and c68) were directed to an epitope that includes residues B20-30. Clones clustered in group 3 (a43, b56, b66 and c56) react with the C-terminal of the B chain; however, their binding was partially inhibited by B1-20. Clone 21 in group 2 also reacted with the C-terminal of B chain, but its binding was neither inhibited by B1-20 nor B20-30 (Additional file 3). An immunohistochemical analysis of paraffin-embedded mouse pancreatic sections showed the staining of β cells by the guinea pig mAb c08 against human insulin (Figure 6F).

## Discussion

The use of ASPCs is best suited to the generation of high affinity mAbs because somatic hypermutation of immunoglobulin genes allows a continual improvement in antibody recognition during the course of PC differentiation. However, the isolation of ASPCs is one of the most challenging types of cell separation due to the scarcity of ASPCs in lymph nodes and blood. Recently, several groups reported a microengraving method for the selection of ASPCs by using an array of on-chip micro wells [5,6]. But the microwell-based method suffers from several problems, including the limited application of this method for species other than humans and mice and the limited numbers of cells that can be analyzed per experiment. FACS, on the other hand, enables the analysis of large numbers of cells at high speeds; however, the isolation of ASPCs by FACS had yet to be established. The main limitation in isolating ASPCs has been the non-specific binding of cells to fluorescently labeled antigens, which is caused by the use multiple antibodies against lineage-specific markers to enrich for PCs. To overcome these limitations, we developed ERIAA for detecting ASPCs from a variety of animals by FACS. Because ERIAA is based on weak cell surface IgG expression and abundant cytoplasmic rough ER, common features of PCs across animal species, a series of antibodies against lineage markers is not required for PC identification. By simply staining lymph node cells with antibodies against cell surface IgG and ER-tracker, we achieved PC identification from humans, mice, rabbits and rats with 70% to 90% purity, representing a 48-fold to 223-fold enrichment of PCs compared with the original lymphocyte populations. Furthermore, the reduction of background noise, due to not using several lineage-specific markers, allowed us to isolate ASPCs with a fluorescently labeled antigen. Unlike hybridoma and antibody display technology, ERIAA does not require a large number of lymphocytes. Because of the abundant PCs and small amount of red blood cells, lymph node is best suited for PCs isolation by ERIAA. Although the number of PCs in blood is significantly lower than that in lymph node, peripheral blood mononuclear cells can be used as a source in the case where human monoclonal antibodies from patients are required. In such a case, the use of ER-tracker in combination with known PC marker such as CD38 provides a high yield of PCs with very high purity. The isolation of ASPCs from spleen by ERIAA is less efficient due to the presence of non-plasma cells with strong ER-tracker signal (unpublished data).

Many protein targets are highly conserved between mice and humans and can therefore be recognized as self-antigens by a mouse host, making them less immunogenic. To avoid this problem of human proteins being recognized as self-antigens in mice, rabbit monoclonal antibody production by hybridomas was developed [19]. Although a variety of animals can act as a potential host for producing mAbs by hybridoma, further application of hybridoma technologies to animals are expected to be limited due to difficulties in establishing a partner cell line for fusion. An alternative method for the generation of monoclonal antibodies by hybridoma is antibody display, which bypasses immune tolerance issues with highly conserved antigens. However, antibodies selected from synthetic repertoires often resulted in low affinity and/or non-specificity compared to antibodies derived from immunized animals. To overcome these limitations, phage antibody libraries were made from immunized animals and monoclonal antibodies with high binding affinity have been obtained [20-24]. Unlike antibody display technology, our method enables the preservation of original heavy-light chain pairings, which is useful in analyzing protective antibodies in infectious disease or identifying autoimmune antibodies in humans.

## Conclusions

Our approach of utilizing ERIAA and high-throughput cloning enables the rapid generation of antigen-specific mAbs from a variety of animals. We verified the advantage of our technology by isolating hundreds of human insulin-specific mAbs from guinea pigs. Our methods will greatly contribute to the isolation of high performance mAbs needed for sensitive diagnostics and therapeutic purposes.

## Methods

### Materials

Antibodies against rat IgG (unconjugated, Dylight 594 conjugated, Dylight 650 conjugated and alkaline phosphatase (AP) conjugated), guinea pig IgG (unconjugated, Dylight 488 conjugated and AP conjugated), rabbit IgG (unconjugated, Dylight 594 conjugated, Dylight 650 conjugated and AP conjugated) and human IgG (Dylight 488 conjugated) were obtained from Abcam. Anti-mouse CD45R-PE, anti-mouse CD38-APC, anti-human CD20-PE and anti-human CD38-APC were obtained from Miltenyi Biotec (http://www.miltenyibiotec.com/en/default.aspx). All antibodies were diluted in 1 × phosphate-buffered saline (PBS) containing 0.5% (w/v) bovine serum albumin (BSA) and 2 mM ethylenediaminetetraacetic acid (EDTA) (PBS-BSA). Human insulin was obtained from Roche Applied Science (http://www.roche-applied-science.com/). Dynabeads® Oligo-(dT)_25 _magnetic beads and ER-Tracker™ Blue-White DPX and primers were purchased from Life Technologies. DNA sequencing was performed using an ABI 373XL DNA sequencer (Life Technologies). The synthetic peptides were purchased from Medical & Biological Laboratories (https://ruo.mbl.co.jp). Restriction enzymes and PrimeStar DNA polymerase with 1 × PrimeStar GC buffer were obtained from Takara Bio (http://www.takara-bio.com/). The framework (FW) regions and complementarity-determining regions (CDRs) were assigned using IMGT/V-QUEST (http://imgt.cines.fr/IMGT_vquest/share/textes/).

### Cell preparation

Animal experiments were approved by the Committee on Animal Experimentation at the University of Toyama. Female ICR mice (6 weeks old), Wistar rats, New Zealand white rabbits and Hartley guinea pigs were purchased from Japan SLC, Inc. (http://jslc.co.jp/). Groups of three animals were used to immunize each antigen. Mice were immunized three times (at intervals of 3 weeks) subcutaneously in the hind footpad with a 50 µl of 50:50 water-in-oil TiterMax Gold adjuvant emulsion (Sigma-Aldrich, http://www.sigmaaldrich.com/) containing 25 µg of egg albumin as an antigen. Rats and rabbits were immunized four times intramuscularly at the tail base with 200 µl of 50:50 water-in-oil TiterMax Gold adjuvant emulsion containing 100 µg of GFP. Guinea pigs were immunized four times intramuscularly at the tail base with a 200 µl of 50:50 water-in-oil TiterMax Gold adjuvant emulsion containing 100 µg of human insulin. At 1 week after the final immunization, the iliac or popliteal lymph nodes were surgically removed and used for the isolation of PCs. Human experiments were performed with the approval of the Clinical Research Ethics Committee at the University of Toyama and Itoigawa General Hospital. We obtained informed consent from all of the subjects. Human lymphocytes were prepared from surgically removed regional lymph nodes from patients with gallbladder and colon cancers.

### Isolation of ASPCs by ERIAA

Cells (1 to 5 × 10^6^/ml) prepared from mouse or human lymph nodes were suspended in 1 ml PBS-BSA and stained with fluorescently labeled antibodies against IgG for 30 minutes at 4°C with rotation. After washing with PBS-BSA, the cells were stained with anti-mouse CD45R-PE and CD38-APC or anti-human CD20-PE and CD38-APC, respectively. The cells were washed twice with PBS and incubated with 1 ml PBS containing ER-tracker (1 µM) for 5 minutes at room temperature. The cell suspension was then diluted with 4 ml PBS and analyzed by FACS. To achieve single-cell sorting of ASPCs, the cells (1 to 5 × 10^6^/ml) prepared from rabbit, rat or guinea pig tissue were suspended in 1 ml PBS-BSA and stained with fluorescently labeled antigen (0.1 µg/ml) and fluorescently labeled antibody against IgG at 4°C for 30 minutes with gentle agitation. After washing with PBS, the cells were stained with ER-tracker as described above. The forward-versus-side-scatter (FSC vs SSC) lymphocyte gate (R1) was applied to exclude dead cells. The PCs (IgG^low ^ER^high^, R2 gate) were further subdivided into fractions according to their binding levels of fluorescently labeled antigens to define the ASPCs (IgG^low ^ER^high ^antigen^+^) and non-specific PCs (IgG^low ^ER^high ^antigen^-^). Single-cell sorting was performed using a JSAN flow cytometer equipped with an automatic cell deposition unit (http://baybio.co.jp/english/top.html) with fluorescently labeled antibodies against IgG monitored in the FL-l (mouse, human and guinea pig) or FL-5 (rabbit and rat) channel, fluorescently labeled antigen in the FL-1 (GFP Dylight 488) or FL-2 (insulin-Cy3) channel and ER-tracker in the FL-7 channel.

### Immunofluorescent analysis

FACS-sorted cells were deposited onto propyltriethoxysilane-coated glass slides (Matsunami Glass, http://www.m-osaka.com/en/exhibitors/231/), fixed with 4% paraformaldehyde and permeabilized with PBS containing 0.1% Triton X-100. The cells were stained with a fluorescently labeled antibody against IgG and fluorescently labeled antigen for 60 minutes at room temperature. The images were captured using an Olympus IX71 fluorescence microscope equipped with a SPOT RT3 digital microscopy camera (SPOT Imaging Solutions, http://www.spotimaging.com/) and compiled using Adobe Photoshop CS5 software. A minimum of 100 cells were counted for analysis in each experiment, and each experiment was performed in triplicate.

### Plasmid construction

DNA fragments encoding the human, rabbit, rat and guinea pig immunoglobulin gamma (IgG), kappa (IgK) and lambda (Igλ) constant regions were amplified using lymphocyte cDNAs as the templates with primers for IgG (IgGC S and IgGC AS), IgK (IgKC S and IgKC AS) and Igλ (IgλC S and IgλC AS), respectively. The amplified DNA fragments digested with *Xho*I and *Not*I were inserted into the corresponding sites of pJON-IgG [14]. The DNA fragments encoding the human insulin A and B chains were subcloned into the *Spe*I/*Xho*I site of the pET42b vector. The human insulin B chain mutants B1-13 and B1-20 were generated using the QuikChange XL Site-Directed Mutagenesis Kit (Agilent Technologies; http://www.genomics.agilent.com/) with wild-type human insulin B chain DNA as the template. The primers used were as follows: B(1-13) forward 5'-TGCGGCTCACACCTGGTGGAAAAACTTGCGGCCGCACTCGAG-3', B1-13 reverse 5'-CTCGAGTGCGGCCGCAAGTTTTTCCACCAGGTGTGAGCCGCA-3', B1-20 forward 5'-GCTCTCTACCTAGTGTGCGGGAAACTTGCGGCCGCACTCGAG-3' and B1-20 reverse 5'-CTCGAGTGCGGCCGCAAGTTTCCCGCACACTAGGTAGAGAGC-3'. Insulin epitope-glutathione S-transferase (GST) fusion proteins were produced in *Escherichia coli *BL21 (DE3) and purified using Superflow Ni-NTA columns (Qiagen, http://www.qiagen.com/default.aspx). The antigens were labeled with DyLight Fluor Labeling Reagents (Thermo Scientific, http://www.thermoscientific.com/) and purified using Bio-Gel columns (BIO-RAD, http://www.bio-rad.com/).

### Molecular cloning of V_H _and V_L _genes from single cells

V_H _and V_L _genes were amplified from sorted single cells as previously described, with some modifications [14]. Single cells were sorted into each well of U-bottom 96-well plates containing 10 µl cell lysis/binding solution (100 mM Tris HCl (pH 7.5), 500 mM LiCl, 1% lithium dodecyl sulfate, 5 mM dithiothreitol and 10 µg oligo-(dT)_25 _magnetic beads). Preparation of the 3'-end homopolymer-tailed cDNA from single ASPCs is performed automatically by a non-contact magnetic power transmission instrument (MAGrahd) [13]. The V_H _and V_L _genes were amplified by 5' RACE PCR using the 3'-end homopolymer-tailed cDNA fragments as the templates. Briefly, the first round of PCR was performed with a forward primer (Nhe polyC S) and a mixture of reverse primers (IgGV AS1, IgKV AS1 and IgλV AS1). PCR was performed using PrimeStar DNA polymerase in 1 × PrimeStar GC buffer with the BIO-RAD MyCycler (35 cycles with denaturation at 95°C for 30 s, annealing and strand elongation at 68°C for 90 s and a final extension at 72°C for 180 s. The resulting PCR mixtures were diluted 1:10 with water and 1 μl of each was used for the second round of PCR. The second round of PCR was performed with a forward primer (Nhe-Eco47) and a respective nested reverse primer (IgGV AS2, IgKV AS2 or IgλV AS2) as described above. The primers used are summarized in Table 1.

**Table 1:** Primers used in this study

Name	Sequence	Application
human IgGV AS1	ACGCTGCTGAGGGAGTAGAGTCCTGAG	5' RACE PCR human V_H _first
human IgGV AS2	AGCCGGGAAGGTGTGCACGCCGCTG	5' RACE PCR human V_H _second
human IgKV AS1	CTTTGGCCTCTCTGGGATAGAAGTT	5' RACE PCR human V_Lκ _first
human IgKV AS2	ACAACAGAGGCAGTTCCAGATTTCAACTGC	5' RACE PCR human V_Lκ _second
human IgλV AS1	TCACDGSYCCCGGGTAGAAGTCACT	5' RACE PCR human V_Lλ _first
human IgλV AS2	AGTGTGGCCTTGTTGGCTTG	5' RACE PCR human V_Lλ _second
rat IgGC S	AGAGACTCGAGTGGAACTCTGGAGCCCTGTCCAGCG	Rat IgG constant gene cloning
rat IgGC AS	CTCTCTGCGGCCGCGGGTCATTTACCCGGAGAGTGGGAGAG	Rat IgG constant gene cloning
rat IgKC S	AGAGACTCGAGGGGCTGATGCTGCACCAACTGTATC	Rat IgK constant gene cloning
rat IgKC AS	CTCTCTGCGGCCGCGTCTAACACTCATTCCTGTTGAAGCTC	Rat IgK constant gene cloning
rat IgGV AS1	GCAGGTGACGGTCTGGCTGGRCCAGGTGCTGGA	5' RACE PCR rat V_H _first PCR
rat IgGV AS2	CTGCAGGACAGCTGGGAAGGTGTGCAC	5' RACE PCR rat V_H _second PCR
rat IgKV AS1	TAACTGTTCCGTGGATGGTGGGAAGAT	5' RACE PCR rat V_Lκ _first PCR
rat IgKV AS2	TCGTTCAGTGCCATCAATCTTCCACTTGAC	5' RACE PCR rat V_Lκ _second PCR
rat IgH cassette S	TGGAACTCTGGAGCCCTGTCCAGCGGTG	Rat IgH cassette amplification
rat IgL_k _cassette S	GGGCTGATGCTGCACCAACTGTATC	Rat IgL_k _cassette amplification
rab IgGC S	AGAGAGCTCGAGTGCCTGGTCAAAGGCTACCTCCCG	Rabbit IgG constant gene cloning
rab IgGC AS	GTGTGTGCGGCCGCGCTCATTTACCCGGAGAGCGGGAGAT	Rabbit IgG constant gene cloning
rab IgKC S	AGAGAGCTCGAGGATCCAGTTGCACCTACTGTCCTCA	Rabbit IgK constant gene cloning
rab IgKC AS	AGAGAGGCGGCCGCTCTAGCAGTCACCCCTGTTGAAGCT	Rabbit IgK constant gene cloning
rab IgGV AS1	GCTGGCTGCTTGAGGTCACGCTCACCAC	5' RACE PCR rabbit V_H _first PCR
rab IgGV AS2	CTGCCGGACGGACGGGAAGGTGCGTAC	5' RACE PCR rabbit V_H _second PCR
rab IgKV AS1	CAGTTGTTTGGGTGGTGCCATCCAC	5' RACE PCR rabbit V_Lκ _first PCR
rab IgKV AS2	GGGTGGTGCCATCCACCTCCCAGGTGAC	5' RACE PCR rabbit V_Lκ _second PCR
rab IgH cassette S	CCAGTTGCACCTACTGTCCTCATCTTCC	Rabbit IgH cassette amplification
rab IgL_k _cassette S	GATCCAGTTGCACCTWCTGTCCTCMTCTTCC	Rabbit IgL_k _cassette amplification
g-pig IgGC S	AGAGACTCGAGTGCCTGGTCAAGGGCTACTTCCCTGA	Guinea pig IgG constant gene cloning
g-pig IgGC AS2	ATCTCCCGGTCTCCGGGTAAATGAGCGGCCGCTCTCTC	Guinea pig IgG constant gene cloning
g-pig IgKC S	AGAGACTCGAGGGGACCAAGCTGGAAATCAAACGGA	Guinea pig IgK constant gene cloning
g-pig IgKC AS	TATATAGCGGCCGCCTAGCACTCGCTCCTGTTGATGGTCT	Guinea pig IgK constant gene cloning
g-pig IgλC S	AGAGACTCGAGTGCCTGGTCAAGGGCTACTTCCCTGA	Guinea pig Igλ constant gene cloning
g-pig IgλC AS	GAGAGAGCGGCCGCTCATTTACCCGGAGACCGGGAGAT	Guinea pig Igλ constant gene cloning
g-pig IgGV AS1	CTTGTCCACCTTGGTGCTGCTGGCCGGGTG	5' RACE PCR guinea pig V_H _first
g-pig IgGV AS2	GACTGAAGGACGGCCGGGAAGGTGTGCAC	5' RACE PCR guinea pig V_H _second
g-pig IgKV AS1	CAGAGCCATCCACCTTCCACTTGACGG	5' RACE PCR guinea pig V_Lκ _first
g-pig IgKV AS2	GAAGAGGGAGATAGTTGGCTTCTGCACACT	5' RACE PCR guinea pig V_Lκ _second
g-pig IgλV AS1	CTGCTGGCCATGTATTTGTTGTCGCTCTG	5' RACE PCR guinea pig V_Lλ _first
g-pig IgλV AS2	AGAAGGAATTCAGGAGACACACCACTGT	5' RACE PCR guinea pig V_Lλ _second
g-pig IgH cassette S	TGCCTGGTCAAGGGCTACTTCCCTGAGC	Guinea pig IgH cassette amplification
g-pig IgL_k _cassette S	GGGACCAAGCTGGAAATCAAACGGAG	Guinea pig IgL_k _cassette amplification
g-pig IgL_λ _cassette S	GAGGAGCTCCAGGACAACAAGGCCAC	Guinea pig IgL_λ _cassette amplification
Nhe polyC S	GCTAGCGCTACCGGACTCAGATCCCCCCCCCCCCCDN	5' RACE PCR first
Nhe-Eco47	CGCTAGCGCTACCGGACTCAGATCCC	5' RACE PCR second
Cassette AS	GGGGGGGGGGGGGATCTGAGTC	Cassette amplification
Joint S	AGAGAAACCGTCTATCAGGGCGATGGC	TS-jPCR
Joint AS	AGAGACCCTTTGACGTTGGAGTCCACG	TS-jPCR

PCR = polymerase chain reaction; RACE = rapid amplification of cDNA ends; TS-jPCR = target-selective joint PCR.

### TS-jPCR

TS-jPCR was performed as previously described [14]. The IgH and IgL DNA cassettes were amplified from the respective pJON plasmids using PCR with primers for IgG (IgH cassette S and Cassette AS), IgK (IgL_k _cassette S and Cassette AS) and Igλ (IgL_λ _cassette S and Cassette AS). The amplified DNA cassettes were purified using S-400 spin columns. The PCR-amplified V gene fragments were joined to their respective DNA cassettes to build linear IgH and IgL genes by TS-jPCR. The primers used were Joint S and Joint AS. The PCR primers are detailed in Table 1.

### DNA transfection

The cognate pairs of IgH and IgL genes produced by TS-jPCR were cotransfected using the FuGENE HD Transfection Reagent (Roche, http://www.roche.com/research_and_development.htm) into 293FT cells grown in 96-well culture dishes. At 3 days after transfection, the cell culture supernatants were analyzed for the secretion of recombinant antibodies. The antibody concentrations and reactivities were determined by enzyme-linked immunosorbent assay (ELISA), as described previously [13]. Large-scale recombinant mAbs were prepared using the FreeStyle™ 293 Expression System (Life Technologies) according to the manufacturer's protocol. The antibodies produced were purified using HiTrap™ Protein A HP columns (GE Healthcare, http://www3.gehealthcare.com/en/Global_Gateway). The protein concentrations were determined spectrophotometrically at 280 nm. The integrity of the produced antibodies was verified using SDS-PAGE with an IgG as the reference.

### Affinity measurement

The wells of an ELISA plate were coated overnight with 50 μl of 50 nM human insulin in 100 mM bicarbonate buffer and blocked with PBS-BSA for 1 h at room temperature. Human insulin (0, 20, 50, 100, 150 and 200 nM) was incubated in PBS with guinea pig mAbs or control IgG (0.5 and 1 nM) overnight at room temperature to reach equilibrium. The antibody mixtures were then transferred to the insulin-coated wells and incubated for 1 h at room temperature. After washing the plate with PBS, the bound guinea pig mAbs were detected with AP-conjugated goat anti-guinea pig IgG. The developed chemiluminescence was quantified using a Tecan GENios microplate reader (TECAN, http://www.tecan.com). The magnitude of light emitted was expressed as relative light units (RLU). Each sample was tested in duplicate. The dissociation constant (K_d_) of the antigen-antibody complexes was determined according to the following equation:

$$Ao/Ao-Ai=1+{\text{K}}_{\text{d}}/{\text{a}}_{\text{o}}$$

where *Ao *and *Ai *are the signal of total antibody incubated in the absence and presence of a given concentration of antigen, respectively, and a_o _is the total concentration of antigen in the antigen-antibody mixture.

### Epitope mapping

Mapping of the antibody epitopes was performed using competitive ELISA. Briefly, epitope-GST fusion proteins (100 nM) and a series of peptides (250 nM) was incubated in PBS with the guinea pig mAbs (1 nM) overnight at 4°C. The peptide/antibody mixture was then transferred to an insulin-coated plate and tested for reactivity against insulin, as described above. The antibody epitopes were also determined by western blot analysis using bacterially expressed GST-fused insulin A chain, B chain and B chain truncation mutants (1-20 and 1-13). The peptides used corresponded to amino acid residues 1 to 10, 6 to 15 and 12 to 21 of the A chain and 1 to 10, 6 to 15, 11 to 20, 16 to 25 and 20 to 30 of the B chain of human insulin.

### Immunohistochemistry

Formalin-fixed, paraffin-embedded mouse pancreatic tissue samples were subjected to double immunohistochemical staining for insulin and glucagon. The primary antibodies used were a rabbit monoclonal anti-human glucagon antibody (Cell Signaling Technology, http://www.cellsignal.com/) to identify α cells and guinea pig monoclonal anti-human insulin antibodies to identify β cells. The insulin signal was visualized with goat anti-guinea pig antibody-AP and the VECTOR Red Alkaline Phosphatase Substrate Kit (Vector Labs, http://www.vectorlabs.com/). The glucagon signal was visualized with goat anti-rabbit IgG Dylight 488. The tissue samples were embedded in ProLong Gold Antifade Reagent with 4',6-diamidino-2-phenylindole (DAPI; Life Technologies), subjected to fluorescence microscopy and analyzed using 2D Deconvolution MetaMorph software (Molecular Devices, http://www.moleculardevices.com/).

## Competing interests

The authors declare they have no competing interests.

## Authors' contributions

NK designed and performed the experiments and wrote the manuscript. MY performed the guinea pig and rabbit experiments and analyzed the data. RF contributed to the rat experiments. MI supervised the work. All authors read and approved the final manuscript.

## Supplementary Material

Additional file 1 **Klotz plots**. Klotz plots of the binding of human insulin to guinea pig monoclonal antibodies (mAbs), as measured using ELISA. a_0_, the concentration of total antigen; A_0_, the chemiluminescence measured for the antibody in the absence of antigen; A_i_, the chemiluminescence measured for bound antibody. The value in the parentheses represents the average of the different guinea pig mAb concentrations (0.5 nM and 1.0 nM). Click here for file

Additional file 2 **Phylogenetic analysis of V_H _and V_Lκ _amino acid sequences of the highly binding guinea pig monoclonal antibodies (mAbs)**. **(A) **Guinea pig mAbs of the same lineage group are boxed and labeled as 1, 2 or 3. **(B) **Sequences of the corresponding V_H _and V_Lκ _amino acid regions of the highly binding guinea pig mAbs. Click here for file

Additional file 3 **Epitope mapping of the highly binding guinea pig monoclonal antibodies (mAbs)**. **(A) **Western blots of epitope-glutathione S-transferase (GST) fusion proteins with guinea pig mAbs. About 0.1 μg of proteins were loaded on 15% SDS-PAGE. Lane 1: GST; lane 2: GST-insulin A; lane 3: GST-insulin B; lane 4: GST-insulin B1-20; lane 5: GST-insulin B1-13. **(B) **Epitope mapping by competitive enzyme-linked immunosorbent assay (ELISA). Excess amount of epitope-GST fusion proteins (10-fold molar excess relative to mAb) or overlapping peptides of human insulin (25-fold molar excess) were used as competitors. Binding of the antibodies to wild-type human insulin without competitors was set as 100%. Each experiment was repeated independently twice, and the mean values are shown. Click here for file
