# Supplementary figures and images for: Rapid production of antigen-specific monoclonal antibodies from a variety of animals
Source: BMC Biol. 2012 Sep 28;10:80. doi: 10.1186/1741-7007-10-80 (PMC3520816; doi:10.1186/1741-7007-10-80)

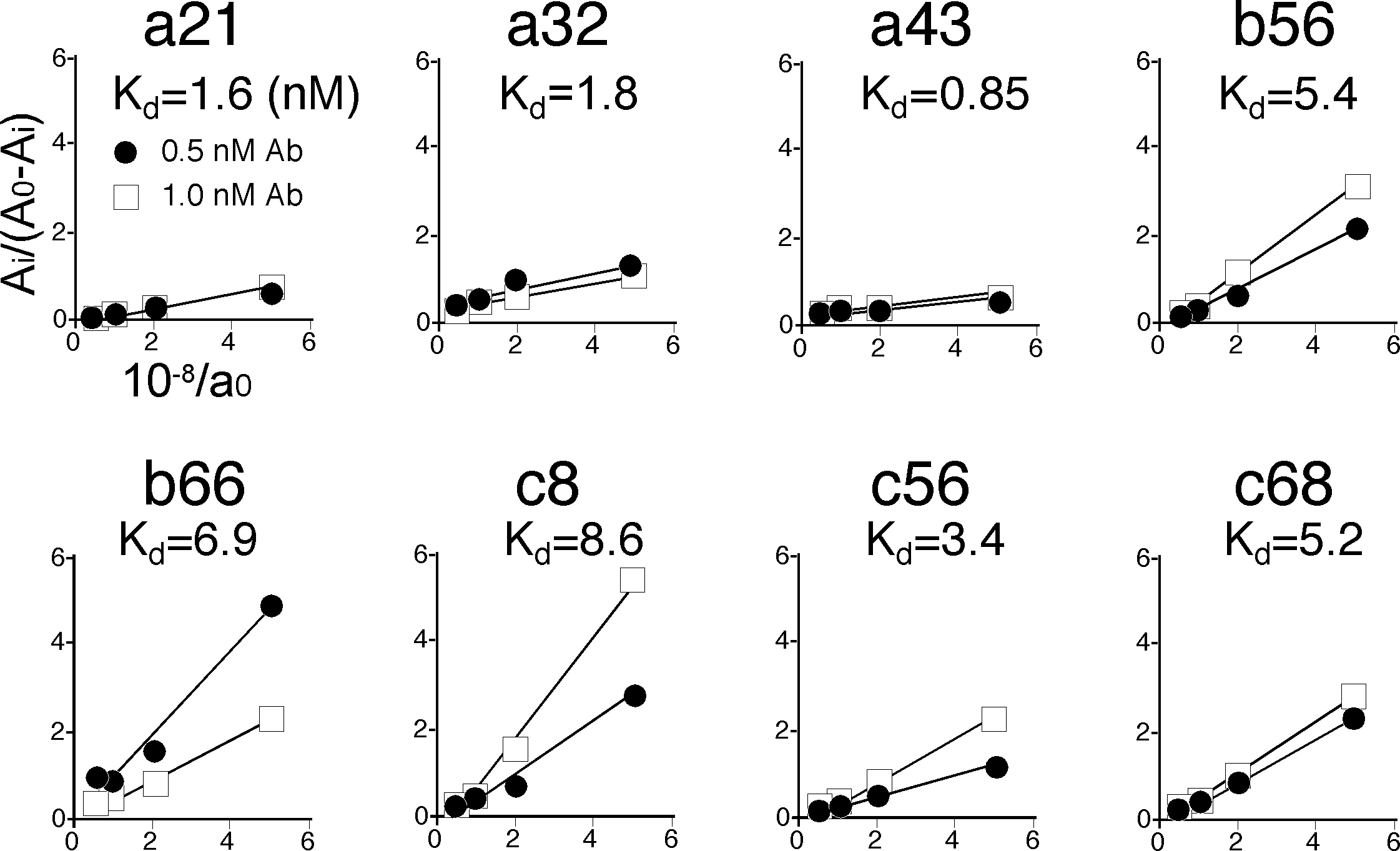

Supplement: Additional file 1 — Klotz plots. Klotz plots of the binding of human insulin to guinea pig monoclonal antibodies (mAbs), as measured using ELISA. a0, the concentration of total antigen; A0, the chemiluminescence measured for the antibody in the absence of antigen; Ai, the chemiluminescence measured for bound antibody. The value in the parentheses represents the average of the different guinea pig mAb concentrations (0.5 nM and 1.0 nM). [file 1741-7007-10-80-S1.TIFF]

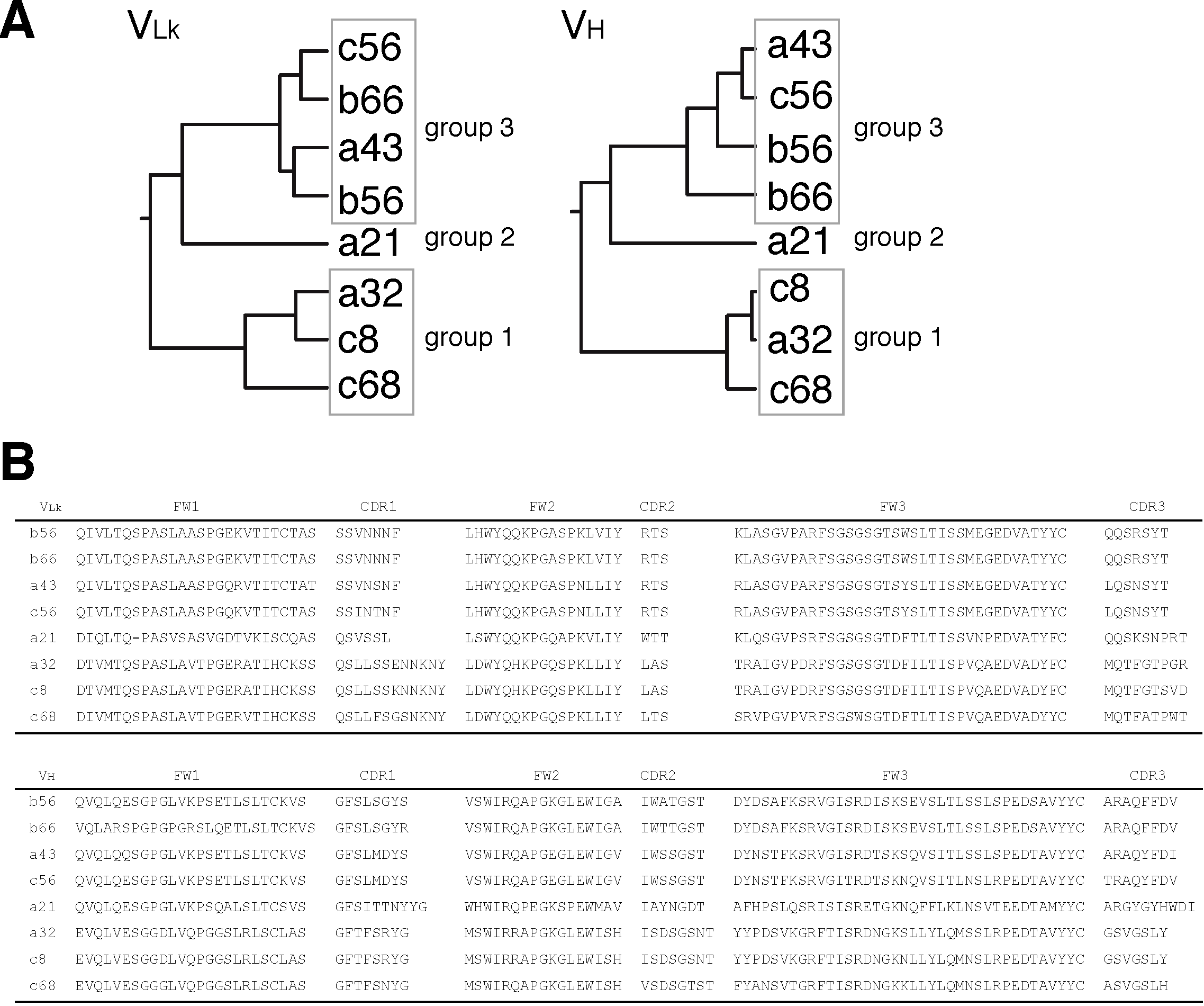

Supplement: Additional file 2 — Phylogenetic analysis of VH and VLκ amino acid sequences of the highly binding guinea pig monoclonal antibodies (mAbs). (A) Guinea pig mAbs of the same lineage group are boxed and labeled as 1, 2 or 3. (B) Sequences of the corresponding VH and VLκ amino acid regions of the highly binding guinea pig mAbs. [file 1741-7007-10-80-S2.TIFF]

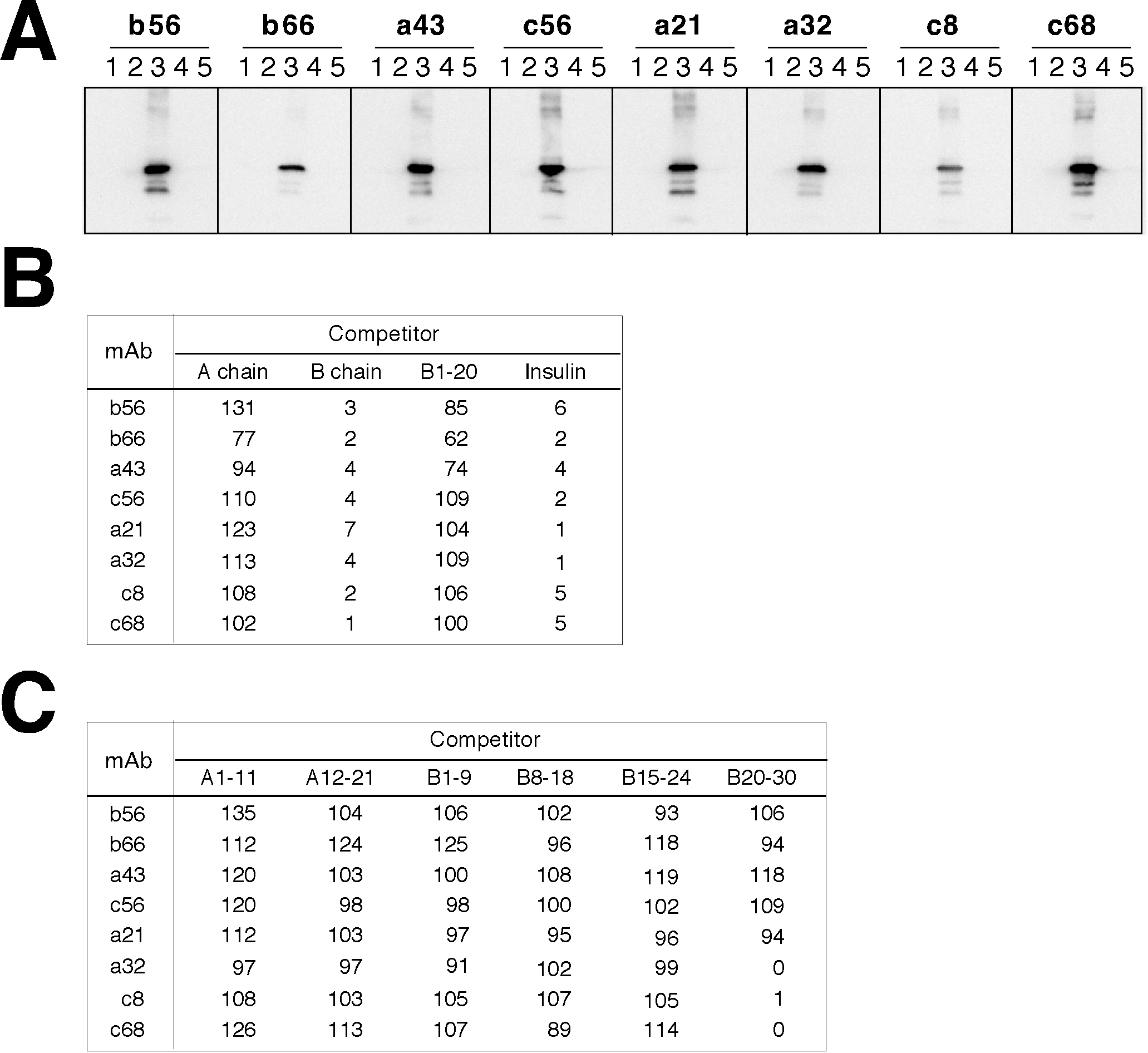

Supplement: Additional file 3 — Epitope mapping of the highly binding guinea pig monoclonal antibodies (mAbs). (A) Western blots of epitope-glutathione S-transferase (GST) fusion proteins with guinea pig mAbs. About 0.1 μg of proteins were loaded on 15% SDS-PAGE. Lane 1: GST; lane 2: GST-insulin A; lane 3: GST-insulin B; lane 4: GST-insulin B1-20; lane 5: GST-insulin B1-13. (B) Epitope mapping by competitive enzyme-linked immunosorbent assay (ELISA). Excess amount of epitope-GST fusion proteins (10-fold molar excess relative to mAb) or overlapping peptides of human insulin (25-fold molar excess) were used as competitors. Binding of the antibodies to wild-type human insulin without competitors was set as 100%. Each experiment was repeated independently twice, and the mean values are shown. [file 1741-7007-10-80-S3.TIFF]
